# $\text {DRTOP}$: deep learning-based radiomics for the time-to-event outcome prediction in lung cancer

**DOI:** 10.1038/s41598-020-69106-8

**Published:** 2020-07-23

**Authors:** Parnian Afshar, Arash Mohammadi, Pascal N. Tyrrell, Patrick Cheung, Ahmed Sigiuk, Konstantinos N. Plataniotis, Elsie T. Nguyen, Anastasia Oikonomou

**Affiliations:** 1 Concordia Institute for Information Systems Engineering, Montreal, QC Canada; 2 Department of Medical Imaging, University of Toronto, Toronto, Canada; 3 Department of Statistical Sciences, University of Toronto, Toronto, Canada; 4 Department of Radiation Oncology, Sunnybrook Health Sciences Centre, University of Toronto, Toronto, Canada; 5 Department of Electrical and Computer Engineering, University of Toronto, Toronto, Canada; 6 Cardiothoracic Imaging Division, Joint Department of Medical Imaging, Toronto General Hospital, Toronto, Canada; 7 Department of Medical Imaging, Sunnybrook Health Sciences Centre, University of Toronto, Toronto, Canada

**Keywords:** Non-small-cell lung cancer, Predictive markers

## Abstract

Hand-crafted radiomics has been used for developing models in order to predict time-to-event clinical outcomes in patients with lung cancer. Hand-crafted features, however, are pre-defined and extracted without taking the desired target into account. Furthermore, accurate segmentation of the tumor is required for development of a reliable predictive model, which may be objective and a time-consuming task. To address these drawbacks, we propose a deep learning-based radiomics model for the time-to-event outcome prediction, referred to as DRTOP that takes raw images as inputs, and calculates the image-based risk of death or recurrence, for each patient. Our experiments on an in-house dataset of 132 lung cancer patients show that the obtained image-based risks are significant predictors of the time-to-event outcomes. Computed Tomography (CT)-based features are predictors of the overall survival (OS), with the hazard ratio (HR) of 1.35, distant control (DC), with HR of 1.06, and local control (LC), with HR of 2.66. The Positron Emission Tomography (PET)-based features are predictors of OS and recurrence free survival (RFS), with hazard ratios of 1.67 and 1.18, respectively. The concordance indices of $68\%$, $63\%$, and $64\%$ for predicting the OS, DC, and RFS show that the deep learning-based radiomics model is as accurate or better in predicting predefined clinical outcomes compared to hand-crafted radiomics, with concordance indices of $51\%$, $64\%$, and $47\%$, for predicting the OS, DC, and RFS, respectively. Deep learning-based radiomics has the potential to offer complimentary predictive information in the personalized management of lung cancer patients.

## Introduction

Despite significant advancements in treatment, lung cancer remains the leading cause of cancer-related mortalities worldwide^1^. Lung cancer is among the most common cancers and, together with breast cancer, includes most of the newly diagnosed cancer cases^2^. Significant recent progress in the biological understanding and tumor heterogeneity of non-small cell lung cancer calls for treatment individualization. Specific clinical endpoints are used in clinical trials to measure the clinical benefit of a specific treatment^3,4^. Although overall survival (OS) remains the gold standard, other clinical endpoints such as recurrence free survival (RFS), distant control (DC), and local control (LC) measure different and significant aspects of the clinical benefit of treatment. Inherent difficulties to assess these clinical outcomes such as the lengthy duration of the follow-up needed until the time of event and the various parameters, unrelated to the primary cancer, affecting the result during follow-up, have led to a surge for developing surrogates that can predict clinical outcomes noninvasively. Recently, radiomics, which is the process of extracting high throughput quantitative and semi-quantitative features from medical images aiming at diagnosis, classification or prediction of outcomes, has attracted much attention, showing promising results^5–15^.

Studies, investigating the relation between radiomics and time-to-event outcomes (e.g., survival and/or recurrence), have mostly focused on hand-crafted radiomics, referring to extracting pre-defined features. Using pre-treatment Computed Tomography (CT) images, Sun et al.^16^ have extracted 339 pre-defined features from the segmented lung tumor volume, to predict the patients’ OS. These features, including shape, size, texture, and intensity statistics, are shown to be predictors of the OS, when going through a set of feature selection and machine learning methods. The prognostic value of hand-crafted radiomics features for OS in lung cancer is also studied by Timmeren et al.^17^, where CT-based extracted features led to a concordance index (a measure of model accuracy) of $69\%$. Khorrami et al.^18^, recently, investigated the correlation of CT-based features with OS and time to progression (TTP) in lung cancer patients treated with chemotherapy, and found a high predictive ability for the extracted features. Although hand-crafted radiomics has shown correlation between imaging modalities and the clinical outcomes, its practical application is limited by the fact that features are pre-defined. Furthermore, hand-crafted radiomics requires the exact segmented region of interest (ROI), being highly dependent on the quality of the segmentation. Obtaining an accurate segmentation is burdensome and subject to inter-observer variability^19^, challenging the reliability of the result.

Considering the potential of radiomics, and at the same time, the limitations associated with hand-crafted radiomics, there has been a recent surge of interest^11,20–23^ in using deep learning, especially Convolutional Neural Networks (CNNs)^24,25^, to extract radiomics features. In deep learning-based radiomics, features are not pre-defined, and do not require the segmented ROI. Therefore, the model can be trained in an end-to-end fashion, with the goal of improving the overall prediction accuracy. Zhu et al.^26^ developed a CNN to predict OS in lung cancer patients and trained the model on pathological images of the lung tumor, leading to a $63\%$ concordance index.

Most of the studies on deep learning-based time-to-event outcome prediction in cancer have focused on features extracted from CT images, which capture only anatomical information. 18-Fluorodeoxyglucose Positron Emission Tomography/Computed Tomography (FDG PET/CT), which combines anatomic data with functional and metabolic information, is the standard of care and has become an integral part of lung cancer staging in clinical practice^27^. The focus of the present work is to propose a novel deep architecture based on staging PET/CT images to predict pre-defined clinical endpoints in a cohort of lung cancer patients before the initiation of treatment.

## Results

### Proposed $\text {DRTOP}$ model for lung cancer time-to-event outcomes prediction

**Figure 1:**
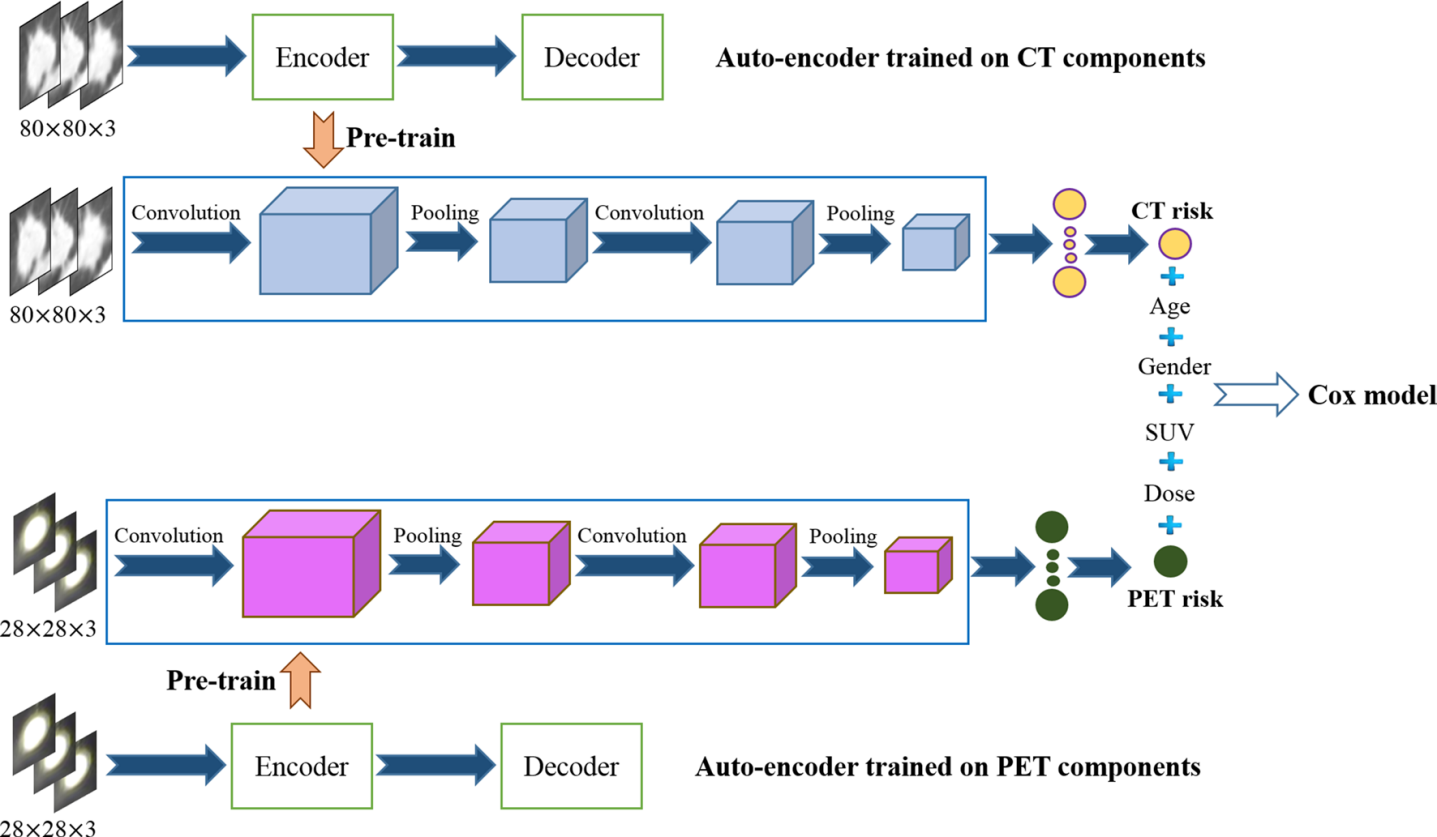
Proposed $\text {DRTOP}$ model, where 3D CT and PET images go through separate networks, which are unsupervisedly pre-trained on an independent dataset. The outputs of the two networks (referred to as the CT risk and the PET risk) are combined with other clinical factors and fed to a Cox PHM.

The proposed deep learning-based time-to-event outcome prediction ($\text {DRTOP}$) model consists of two parallel CNNs, one of which is trained on CT components of the PET/CT, and the other on the PET components. Based on the annotation provided by a thoracic radiologist (A. O.), all images are cropped to fit the tumor, and zero padded (black pixels added to the margins) to have a fixed size. Both CNNs take 3D inputs, which means, for each tumor, we take not only the middle slice, but also the two immediate neighbors. Furthermore, both CNNs are pre-trained using two separate Auto-encoders. The outputs of the CNNs, referred to as the “CT risk” and the “PET risk”, which are trained to maximize the Cox partial likelihood, are fed to a Cox proportional hazards model (PHM), along with four clinical factors, namely age, gender, maximum standardized uptake value (SUV), and radiation dose. The Cox PHM’s coefficients are, also, calculated with the goal of maximizing the Cox partial likelihood. Finally, based on the trained model, concordance indices are computed. The schematic of our proposed $\text {DRTOP}$ model is shown in Fig. 1. The complete model is trained four times, for four different time-to-event outcomes, namely OS, RFS, DC, and LC.

### Performance of the proposed $\text {DRTOP}$

We have evaluated our proposed $\text {DRTOP}$ model based on an in-house dataset, consisting of 132 lung cancer patients, who underwent staging PET/CT before initiation of treatment. The patients are censored when they die or are lost to follow-up. We have trained the $\text {DRTOP}$ model, with the aim of predicting the OS, RFS, DC, and LC. The Cox PHM is trained using 6 predictors (covariates), which are CT risk, PET risk, age, gender, SUV, and radiation dose. The significance of these predictors is tested, and only the significant ones are included in the final model to calculate the concordance index. The results are presented in Table 1.

The OS can be predicted by CT risk, PET risk, and age, where PET risk with the HR of 0.67 has a negative impact (protective effect) on the OS, CT risk with the HR of 1.35 has a positive (an increased risk) impact, and the impact of age is relatively small. The obtained HRs can be interpreted as follows: (1) one unit increase in the CT risk predictor variable results in an increase in the risk of the event occurring by $35\%$; (2) Increasing the PET risk by one unit leads to a $33\%$ risk reduction, and; (3) One year increase of the age can only increase the hazard by $1\%$. The concordance index of $68\%$ shows that the three predictors are capable of providing a satisfying ranking of the patients, with regards to the OS.

**Table 1:** Results from the proposed $\text {DRTOP}$ model. HR stands for hazard ratio (exponent of the obtained coefficient).

Clinical outcome	Significant predictors	Concordance index (%)
OS	CT risk (HR: 1.35, p-value: $<0.005$), PET risk (HR: 0.67, p-value: $<0.005$), Age (HR: 1.01, p-value: 0.02)	$68$
RFS	PET risk (HR: 1.18, *p*-value: $<0.005$), SUV (HR: 1.13, *p*-value: $<0.005$)	$40$
DC	CT risk (HR: 1.06, *p*-value: $<0.005$), SUV (HR: 1.09, *p*-value: 0.02)	$63$
LC	CT risk (HR: 2.66, *p*-value: 0.03)	$37.5$

Significant predictors are obtained based on an F-test, with a significance level of 0.05. Concordance index is calculated on the test set.

### Performance of the hand-crafted radiomics

Out of 42 PET and CT hand-crafted radiomics features, calculated as suggested by a previous study^28^, 18 features (principal components) are extracted using the principle component analysis (PCA). These features, together with SUV, age, gender, and radiation dose, are fed to a Cox PHM to explore predictive models for the specific time-to-event outcomes. Table 2 illustrates the obtained results. Radiomics (PC2) is the only predictor of the OS. Radiomics (PC1 and PC2), together with SUV, contribute to the prediction of RFS and DC. Neither hand-crafted radiomics nor clinical factors can predict the LC. A failed prediction for LC, using hand-crafted radiomics, does not mean a c-index of 0. It means that the c-index is not calculated because the Cox PHM has not found any significant predictors for the LC, where significance is assessed using an F-test. In other words, any calculated c-index, in this case, is not reliable and can be the result of a random model. The c-index is not necessarily an indicator of the predictors’ performance, when they fail to statistically predict enough of the variability in the outcome. If no predictors are found for the model then the hazard function is equal to the baseline hazard. In the case of Cox PHM, the baseline hazard is not estimated as it is a semi-parametric approach, which was designed specifically to benefit from NOT having to estimate the baseline hazard.

**Table 2:** Results obtained from hand-crafted radiomics.

Clinical outcome	Significant predictors	Concordance index
OS	PC2 (HR: 0.44, p-value: 0.02)	$51\%$
RFS	PC1 (HR: 1.57, p-value: 0.02), PC2 (HR: 0.37, p-value: $<0.005$), SUV (HR: 1.14, p-value: $<0.005$)	$47\%$
DC	PC1 (HR: 1.58, p-value: 0.03), PC2 (HR: 0.33, p-value: $<0.005$), SUV (HR: 1.14, p-value: $<0.005$)	$64\%$
LC	-	NA

As there is no significant predictor for the LC, Concordance index is not calculated.

### Comparison of the DRTOP and hand-crafted radiomics

**Figure 2:**
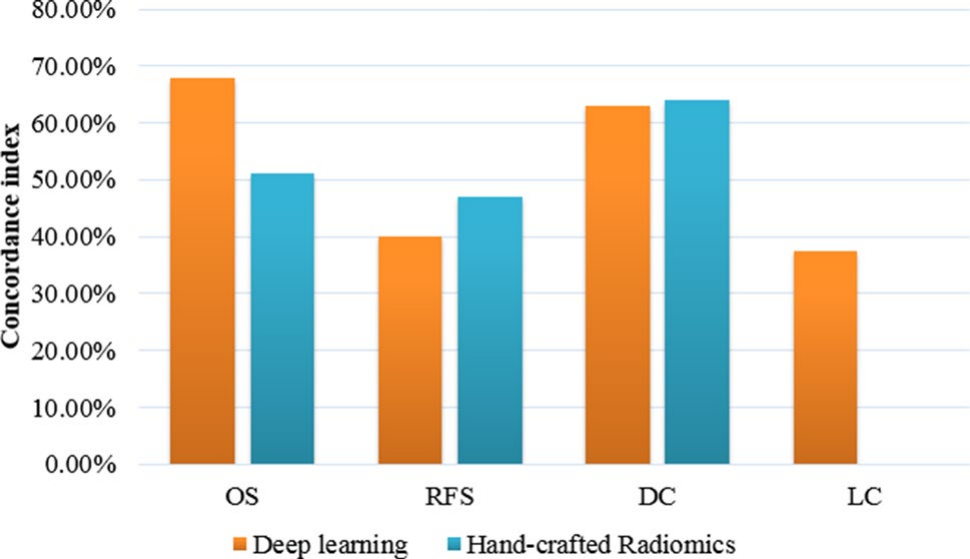
Comparison between our proposed $\text {DRTOP}$ model and hand-crafted radiomics, based on the concordance index.

Figure 2 shows the comparison between the concordance indices obtained from the hand-crafted radiomics and the proposed $\text {DRTOP}$ model. The performance of the proposed model is better than the hand-crafted method, in predicting the OS. Although both methods fail to provide a satisfying result for predicting the RFS, the hand-crafted radiomics has a slightly better performance. The two methods are on a par with each other, in predicting the DC, and in the case of the hand-crafted method, no significant variable is identified to predict the LC. We also attempted to predict the time-to-event outcomes, based on the combination of hand-crafted and deep learning-based features. However, this did not improve the predictive ability of the model.

### Kaplan–Meier curves and cut-off values

**Figure 3:**
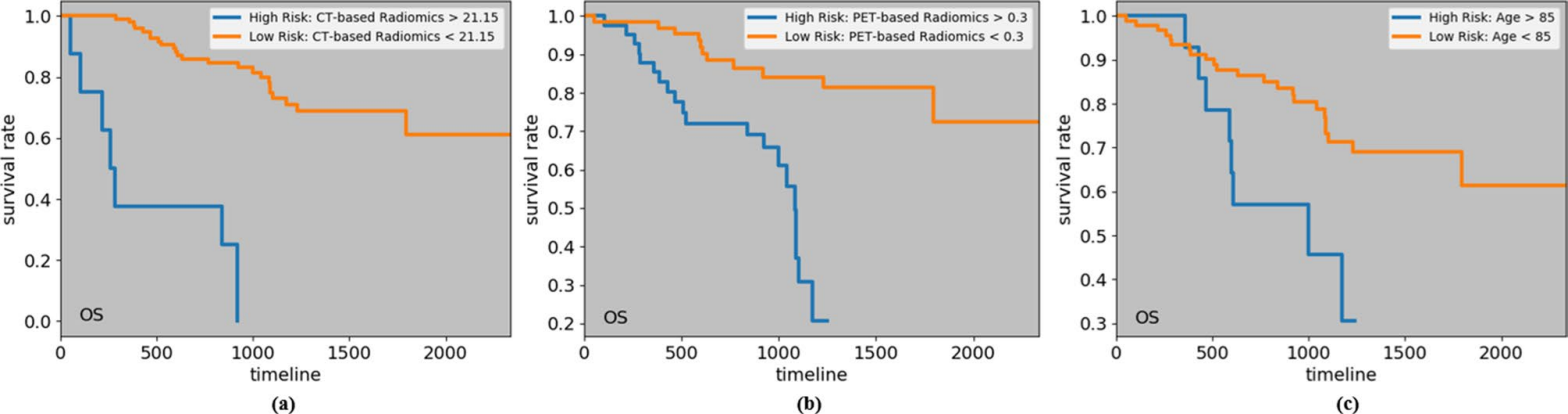
Kaplan–Meier curves associated with the OS, with respect to (**a**) CT risk, (**b**) PET risk, and (**c**) age. Cut-off values to determine the low and high-risk groups are obtained from a logrank test. All predictors, when considered independently, have positive correlations with the OS.

**Figure 4:**
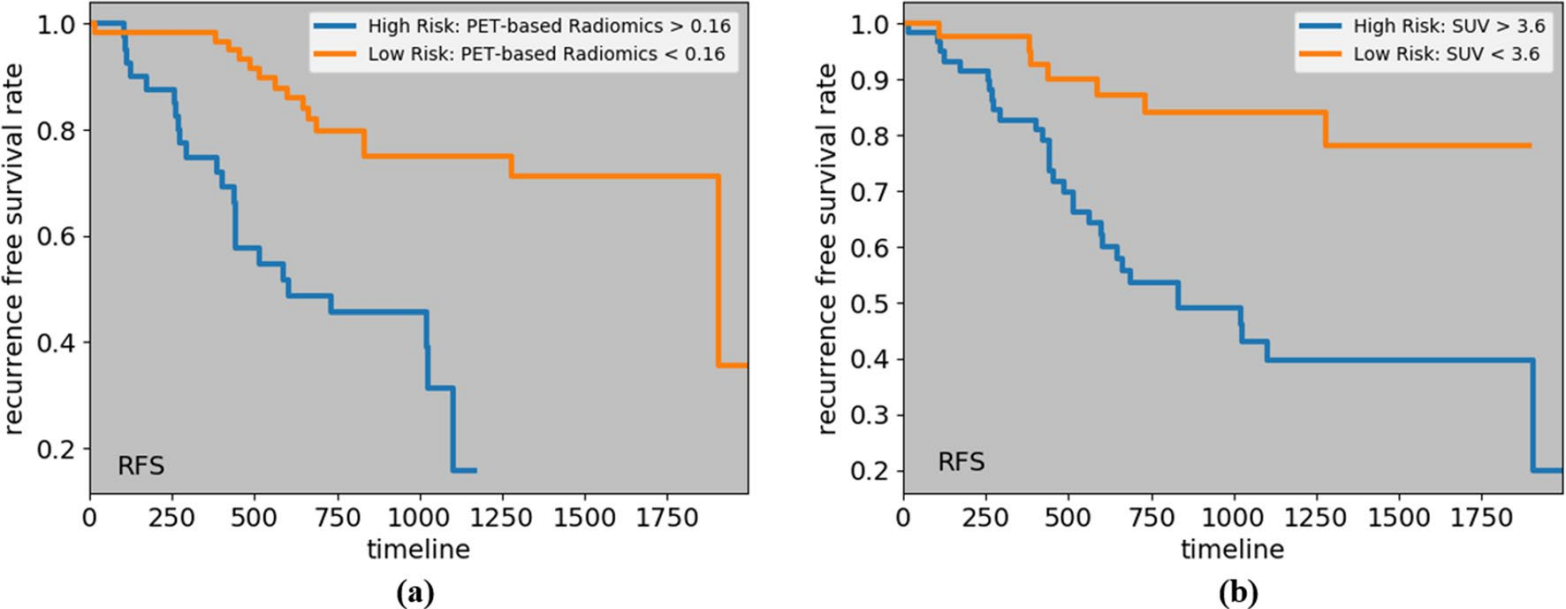
Kaplan–Meier curves associated with the RFS, with respect to (**a**) PET risk, and (**b**) SUV.

**Figure 5:**
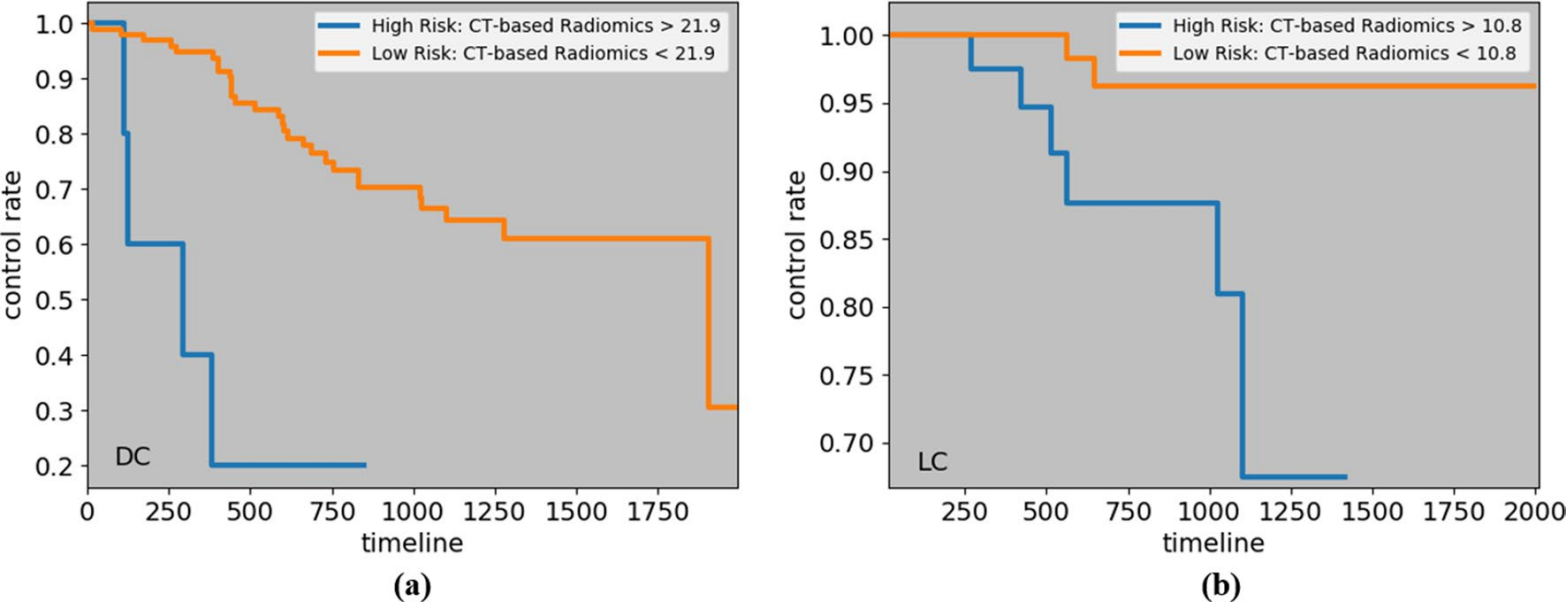
Kaplan–Meier curves corresponding to (**a**) DC, and (**b**) LC, with respect to the CT risk, where cut-off values are determined by a logrank test.

To visualize the impact of a variable on the survival function of different groups, such as low-risk and high risk ones, Kaplan–Meier estimation technique^29^ is utilized. The cut-off value to identify these two groups is often calculated based on a logrank test^30^, that tries to maximize the survival or recurrence difference between the two groups. Considering the significant predictors of the four time-to-event outcomes, we have computed the cut-off values, and obtained the low and high-risk groups, as shown in Figs. 3, 4 and 5. The cut-off values to identify low and high-risk groups from CT risk, PET risk, and age (in years) for OS are 21.15, 0.3, and 85, respectively. In other words, a patient having a CT risk higher than 21.15, and/or a PET risk higher than 0.3, and/or age higher than 85 is considered a high-risk patient, and has a lower chance of survival compared to a low-risk patient. It should be noted that while PET risk is associated with a hazard ratio of less than one in the DRTOP model, meaning that it has a negative impact on the outcome when combined with other factors, it has a positive impact when it is the only predictor taken into account. The cut-off values obtained from the PET risk and SUV for the RFS, are 0.16 and 3.6, respectively, and the cut-off values obtained from the CT risk for DC and LC are 21.9 and 10.8, respectively.

### Random survival forest (RSF) analysis

Our results demonstrated that, based on an RSF model, recurrence free survival (RFS) can be predicted with a concordance index of $64\%$, while, based on the variable importance (VIMP) values presented in Table 3, all predictors, except the radiation dose, show predictive importance.

**Table 3:** Variable importance values obtained from the RSF, for recurrence free survival prediction.

Variable	CT risk	PET risk	Age	Gender	SUV	Radiation dose
VIMP	60.72	71.36	0.59	6.73	53.90	$-4.82$

The negative value means no predictive importance.

**Figure 6:**
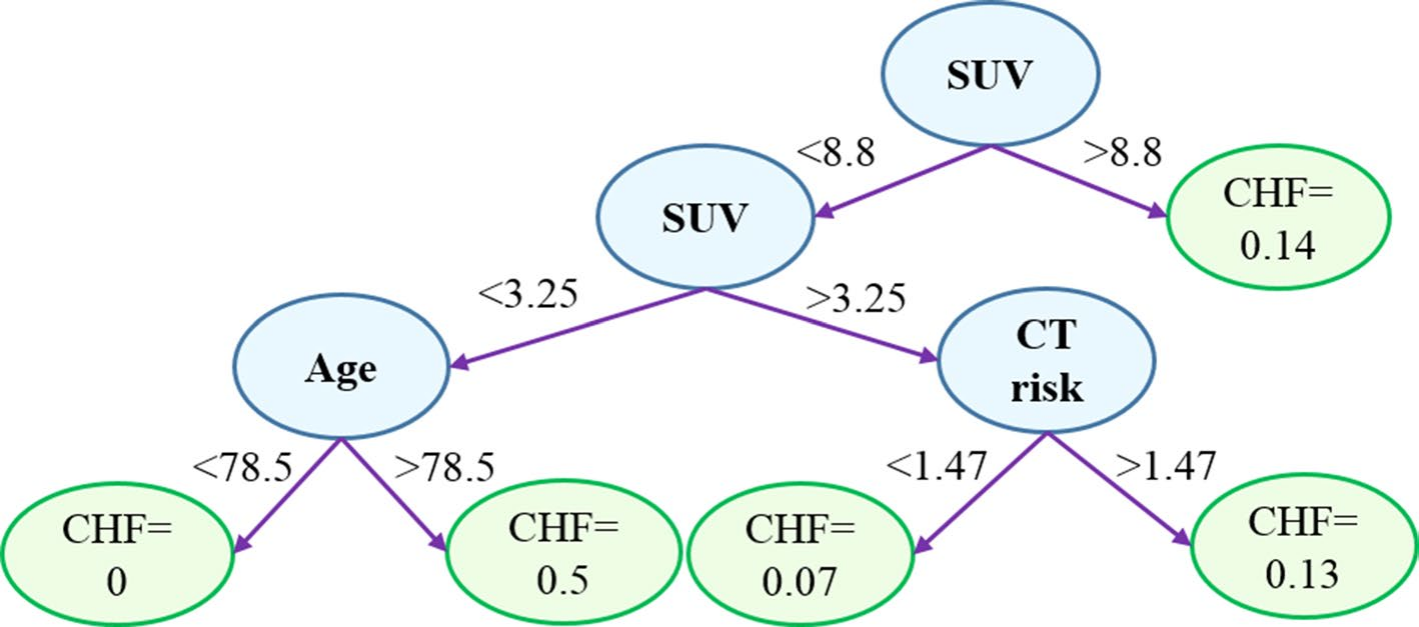
One of the trees obtained from the random survival forest (RSF) to predict the recurrence free survival (RFS). Following this tree, one is able to obtain the cumulative hazard function (CHF), for each patient, at a desired time point. Based on the CHFs, the survival can also be calculated.

Figure 6 shows one of the obtained trees from the RSF. Cumulative hazard function (CHF) is calculated for all the terminal nodes, and all the unique time points. However, only the CHF associated with the first event time is shown in this figure. It should be noted that the left terminal node is associated with a CHF of zero, meaning that no recurrence event has been observed at this node. The RSF model, however, did not reveal any important predictor for the LC, and the concordance index could not be improved. Likewise, the RSF did not improve the accuracy of predicting the OS and DC, compared to the Cox PHM.

The 2-year risk score for RFS can be estimated by summing over the CHF values up to 2 years, obtained at discrete time points. This score can, specially, be used to compare the 2-year RFS risk scores of patients. For instance, for two patients, one of which is censored after three years and 4 weeks, and the other has experienced the event of recurrence after one year and 3 weeks, the risk scores obtained from the RSF are 7.5 and 11.27, respectively.

### Interpretability of the deep learning-based features

**Figure 7:**
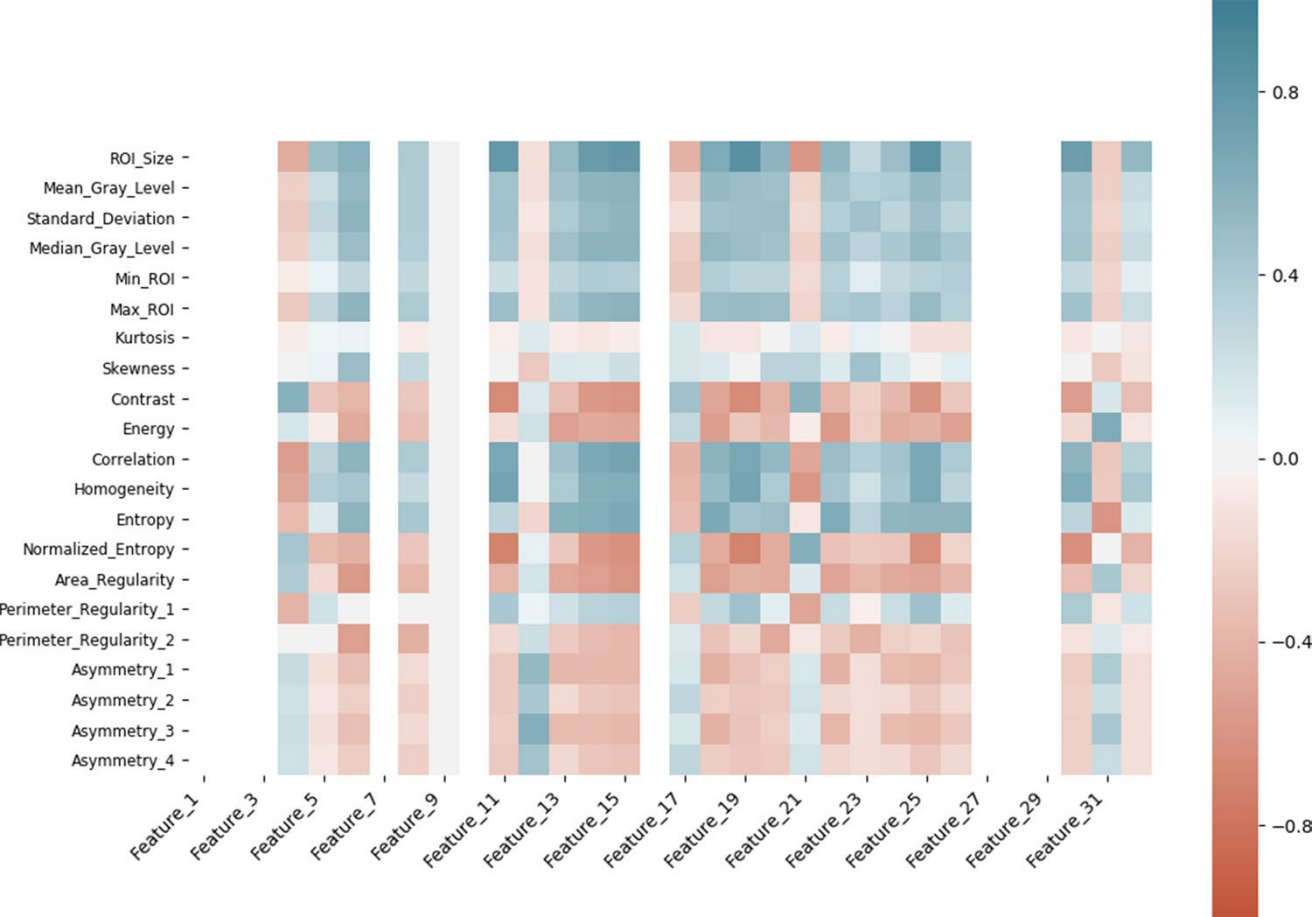
Correlation of the hand-crafted radiomics features extracted from PET images, with the deep learning-based features extracted from layer before the output layer of the model, trained on PET images.

**Figure 8:**
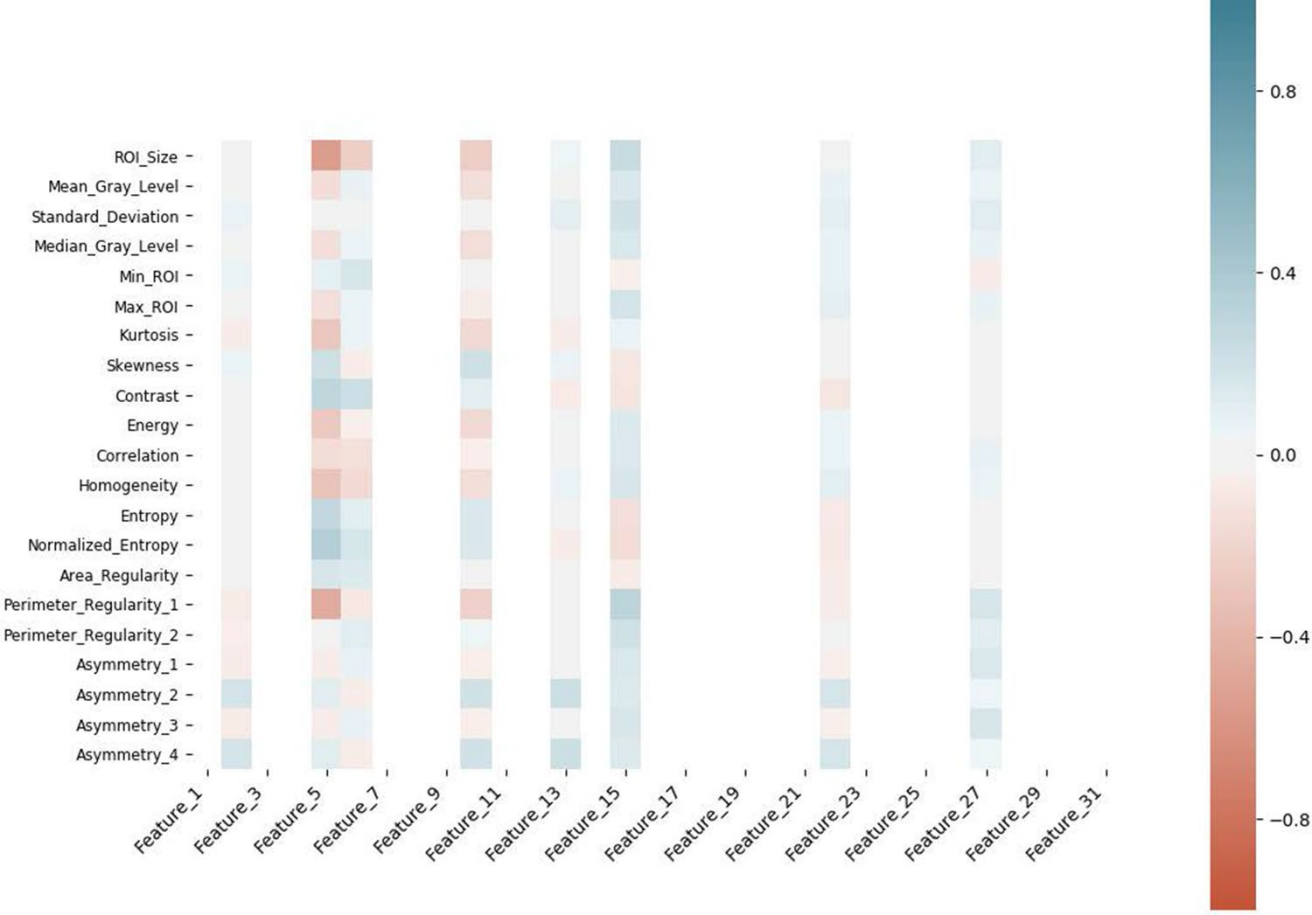
Correlation of the hand-crafted radiomics features extracted from CT images, with the deep learning-based features extracted from layer before the output layer of the model, trained on CT images.

To enhance interpretability of the extracted deep features (make them more tangible), we have conducted correlation analysis between the features extracted from the layer before the final layer in the DRTOP model and hand-crafted features, as shown in Figs. 7 and 8 . In these heat maps, blue and red colors show positive and negative correlation, respectively. The darker the color, the stronger the relation. As it can be inferred from Figs. 7 and 8 , features associated with the PET-risk are highly correlated with hand-crafted features extracted from PET images. The ones associated with the CT-risk, also, show correlation with some hand-crafted features extracted from CT images, although the correlation is not as strong as it is with the PET-risk.

## Discussion

In this work, we propose a novel deep architecture, referred to as deep learning-based radiomics for time-to-event outcome prediction ($\text {DRTOP}$), consisting of two parallel CNNs, one of which was trained based on the CT component, and the other based on the PET component of the PET/CT. The output of the two models (referred to as CT and PET risks), together with clinical parameters such as standardized uptake value (SUV), are fed to a Cox proportional hazards model (PHM)^31^, to predict the time-to-event outcomes. The correlation between SUV and time-to-event outcomes has been previously studied, and it has been shown that SUV is of prognostic value for overall survival (OS)^32^, local control (LC)^33^, and recurrence free survival (RFS)^34^. The SUV is, however, incapable of predicting the outcome, independently^35^. To the best of our knowledge, this is the first time-to-event study that applies a deep learning method to the PET/CT images for staging lung cancer. Moreover, unlike most of the previous studies, which are limited to predicting the OS, our study explores the prediction of RFS, LC, and DC, which are of high clinical value.

**Figure 9:**
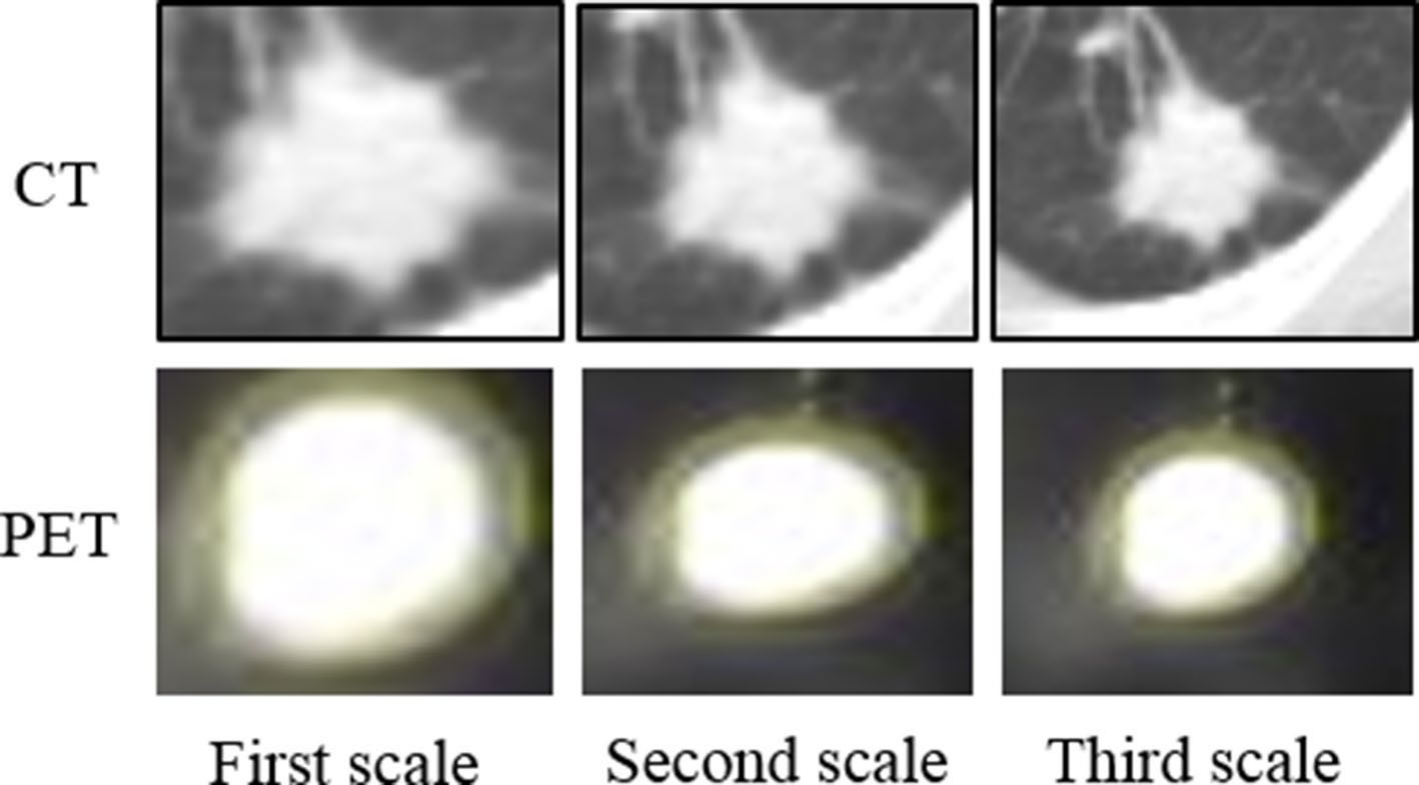
The CT and PET components cropped from three scales. These scales are stacked together and fed to the network.

Generally speaking, it is difficult to directly compare our study with previous works, as models are developed based on different datasets. Next, we focus on highlighting the differences between the proposed DRTOP architecture and previous relevant studies. Considerably lower than the obtained concordance index (c-index) of $68\%$ using the proposed DRTOP model, the CNN model proposed by Zhu et al.^26^ reaches a c-index of $62.9\%$ in predicting OS in lung cancer patients, utilizing pathological images, which capture different information, compared to PET/CT images. Furthermore, the clinical parameters, such as SUV, and their predictive importance, are not considered in their study. The deep learning-based OS prediction model, developed by Wang *et al.*^36^, reaching a c-index of $70\%$, also differs from DRTOP, in that multi-scale CT slices are utilized. Multi-scale input refers to including not only the tumor region itself, but also the surrounding tissues. Features extracted from the ROI (tumor) are not the only features that might influence the outcome. Studies^18^ have shown that the tissues surrounding the tumor may also play a role in predicting the outcome. To be able to compare the predicted OS, using the DRTOP model, with the study by Wang et al., we modified the DRTOP framework to account for multi-scale inputs. To achieve this, we cropped the CT and PET slices from three different scales, shown in Fig. 9, where the first scale completely fits the tumor region, and the second and third scales are constructed by adding 10 and 20 pixels to each side of the tumor boundary, respectively. The three scales are stacked together, to form a 3-channel input, for both CT and PET scans. Other details of this modified architecture are similar to the DRTOP framework. The c-index, however, increases from $68\%$ to $73\%$, which shows the importance of including multi-scale inputs. Our future plan is to study the impact of the surrounding regions of tumor on other time-to-event outcomes, including RFS, DC, and LC. Furthermore, investigations are required to identify which scale has a higher contribution to the final prediction, and what extent of the surrounding tissue suits the time-to-event outcome prediction. We would like to point out that although a multi-scale setting might improve the overall performance, it comes with extra computational and processing cost. Another direction for future research is to find the trade-off between performance and computational cost. Finally, compared to the hand-crafted approach^28^, the proposed DRTOP framework leads to a better performance in predicting the OS, increasing the c-index from $51\%$ to $68\%$, because deep learning model is trained on its own, on the entirety of the image, as opposed to hand-crafted radiomics that are based on certain characteristics of the image.

PET risk and SUV are the only significant predictors of the RFS. However, there is much variability in the RFS that cannot be explained by the identified predictors, based on the Cox PHM. To the best of our knowledge, the prediction of lung cancer RFS, using deep learning, has not been previously investigated. The DC can be predicted by CT risk and SUV, leading to a concordance index of $63\%$. Deep learning-based DC prediction has been recently investigated by Xu et al.^37^, where serial CT images are utilized to update the prediction, after each follow-up. This study, however, fails to provide high accuracy, having only the pre-treatment scans, without any follow-ups, which is the main goal of our work. The c-index using pre-treatment images only, reported by Xu et al., is $58.9\%$ for 1 year distant control, and $58.5\%$ for 2 year distant control. DRTOP and hand-crafted radiomics are almost on a par with each other in predicting DC. While CT risk remains the only significant predictor of the LC, it does not lead to a satisfying concordance index. This means that there may be other factors influencing the LC. Hand-crafted radiomics completely failed to find predictors for the LC, as these features are extracted without considering the final goal, and there is no guarantee that they can contribute to the prediction.

It should be noted that all the results are reported based on the test set ($20\%$ of the whole dataset), and the low c-index obtained for LC, using the proposed DRTOP model, does not indicate a poor performance on the training set. In fact, our model was able to fit the training set and reach a high concordance index of $75\%$. Nevertheless, it failed to generalize well for the test set. This is the reason why the performance of the LC prediction was low. Increasing the number of patients may improve the performance.

As the Cox PHM is a semi-parametric model, thus, restricted to a predefined class of functions, we hypothesized that the poor performance may be due to an insufficiently met relationship between the predictors and the outcomes (RFS and LC). In other words, to ensure the appropriateness of the Cox PHM, the proportional hazards assumption must be met, which is not always the case. We, therefore, replaced the Cox PHM with a random survival forest (RSF)^38^, which does not make this assumption, and calculated the importance values of the predictors, along with the final concordance indices. Our results demonstrated that, based on the RSF model, recurrence free survival (RFS) can be predicted with a concordance index of $64\%$, while all the predictors, except the radiation dose, showed high predictive value. Although a Cox PHM cannot predict the RFS, a non-parametric model can better explain the relation between the predictors and the outcome. Furthermore, although it is computationally expensive to calculate the cumulative hazard and absolute risk from the Cox PHM, as the baseline hazard is almost impossible to estimate, the RSF can be more easily used to provide the risk score. The RSF, however, may be biased, in the sense that it favors the variables with many split-points^39^. Variables with more split-points have a higher influence on the prediction error, and as such, they may be given more importance value.

Stability and reliability of the hand-crafted radiomics depend on the segmentation provided by the experts. Hand-crafted features may vary based on the initial segmentation, when different experts annotate even the same ROI, leading to “inter-observer variability”^19^, and reducing the reliability of the predicted outcomes. We would like to clarify that deep learning-based solution does not necessarily require information about the exact tumor boundaries. In other words, a rough estimation of the location of the tumor is enough to determine the tumor boundary box and accordingly crop the image. The cropped image (instead of exact segmented tumor) is the input to the deep learning model. It is a common practice in different deep learning-based radiomics applications^40^ to replace the exact contouring of the tumor with a single point placement, which in turn minimizes both the human effort and the inter-observer variability. In hand-crafted radiomics, on the other hand, features are extracted from the exact tumor contour, and thus the features are highly sensitive to the segmentation, introduced as a source of human bias^40^. In our study, the input to the deep learning model is a rough boundary box around the tumor. Nevertheless, to compare our results with hand-crafted radiomics, we have annotated the tumors and extracted a set of pre-defined features, that have, consequently, gone through a principle component analysis and Cox PHM. As in this work, our annotations are provided by one expert, we were unable to investigate and compare the inter-observer variability of the hand-crafted and deep learning-based survival and time-to-event analysis, and this can be considered as one of the limitations of our study. Other limitations of the study are the small number of cases included, the lack of external validation and the retrospective nature of the study.

Radiomics use data characterization algorithms that are automatically extracted from delineated ROIs in order to obtain a mineable feature space. The main objective of Machine learning (ML) algorithms, which learn by inference from a dataset, is to produce a model capable of classification/ prediction from selected known data and in doing so improve the decision-making process as it can encompass a higher number of parameters than humans. Deep learning, as an advanced ML technique, allows researchers to model non-linear/ non-parametric decision surfaces while minimizing over-fitting in a very high dimensional space, which is one of the challenges in radiomics. By exploring and combining both ML and traditional radiomics methodologies (as we have done in this study) we will inevitably get closer to the goal of introducing these models to the radiologist’s daily workflow. More specifically, although hand-crafted radiomics and deep learning-based radiomics aim at the same final target—namely predicting survival and recurrence in lung cancer—they have very different but also complimentary approaches and one cannot entirely replace the other. Hand-crafted radiomics use specific known characteristics of the image that may be interpretable and meaningful according to their biologic identity, while deep learning-based radiomics is a “black box” and uses the image as an entirety without providing information about which characteristics of the image contribute to the result and therefore is not directly interpretable. On the other hand, features extracted in hand-crafted radiomics are pre-defined, whereas they are optimized for the problem at hand in deep learning-based radiomics. Therefore, hand-crafted radiomics provides insight to the radiologist and treating physician as to what individual characteristics of the image are associated with survival/recurrence and how to translate them into a treatment plan, while deep learning-based radiomics provides the radiologist, the treating physician, and possibly the patient with an overall risk of death/recurrence with more certainty but less interpretable information. When comparing two different data driven methodologies for modeling the ability of an image to predict time-to-event outcomes, one can expect the results to differ in some respects, especially when the study sample size is limited. The important point is that the interpretation of the results does not change significantly. The choice of which methodology is superior is, in this case, a moot point as we are suggesting that both methodologies offer complimentary useful findings. Deep learning clearly has a role to play in image analysis now and in the future. We, by way of this study, are expressing one of many possible applications of deep learning which we believe enhance the radiologist’s ability to interpret and predict survival from the medical image. The combination of the two approaches may yield higher level of certainty about the overall risk and at the same time provide deeper insight about the process, which could translate into improved personalized treatment for the patient.

In conclusion, the proposed deep learning-based model on staging PET/CT images predicted the overall survival, recurrence free survival and distant metastasis in lung cancer patients. The comparison with hand-crafted radiomics showed that the deep learning model had a relatively better performance compared to the hand-crafted approach. While hand-crafted radiomics will continue to foster medical imaging research and give new insights about individual characteristics of medical images in patients with lung cancer, the combination of the two approaches may prove to be the future for clinical application. It should be noted that despite all the advancements in radiomics, there is still a long way until it is utilized as a stand-alone decision making tool^11^. Challenges include the difficulty of acquiring rich amounts of training samples, considering the privacy issues and lack of homogeneous cohorts of patients, the difficulty of obtaining ground truth, unbalanced data, and image noise. The proposed model, however, can assist the radiologist with having a pick on factors and variables that are not available to the unaided human eye. In other words, deep learning-based radiomics may add complimentary predictive information in the personalized management of lung cancer patients.

## Methods

This project is part of a study approved by the Research Ethics Board of Sunnybrook (REB) Health Sciences Centre (study ID: 337-2018). Furthermore, all methods were carried out according to relevant guidelines and regulations. The Sunnybrook REB determined that an informed consent form was not required for this study.

### Data description and pre-processing

**Figure 10:**
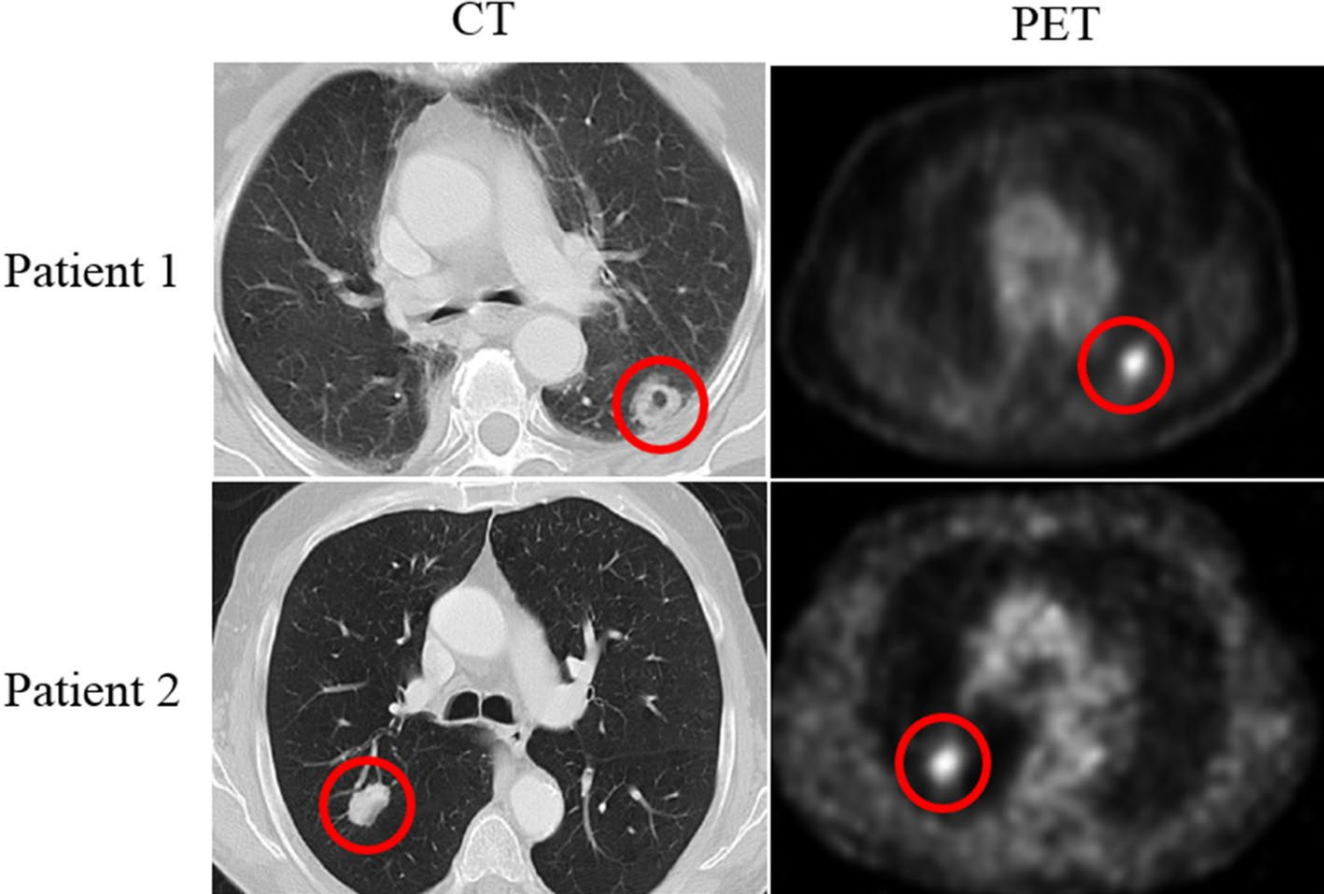
The CT and PET components of the PET/CT, for patient 1 and patient 2, show the tumors in the superior segment of the left lower lobe and the right lower lobe respectively. CT and PET images for each patient are captured at the same level.

The in-house dataset we used in this work consists of 132 lung cancer patients (65 women, 67 men) with an average age of 74.65 (range: 52–92), having 150 lung tumors in total, (treated between April 2008 and September 2012), who underwent staging pre-treatment PET/CT. Tumors visible in both CT and PET components are annotated by a thoracic radiologist, with 18 years of experience in thoracic imaging (A. O.), using an in-house software described in Reference^28^ as follows: Each lesion was contoured on every sequential slice that was visible on CT as increased homogeneous or ground glass density compared to surrounding normal lung parenchyma. Attention was made so that volume averaging areas, and adjacent vascular structures were not included in the regions of interest. The segmentation/contouring of the lesions on the PET images was performed manually on all the sequential images showing increased FDG uptake in the corresponding area of the tumor, which was either the same area covered on the equivalent CT images or slightly smaller. Figure 10 shows the observed tumor for two patients on the CT and PET component, at the same level. Other characteristics that were entered and assessed in the analysis include age, gender, SUVmax, and radiation dose (prescribed biological effective dose). All the patients had early stage lung cancer (N0M0) and were treated with a specific high dose and focused radiotherapy method (SBRT)^28^. Post-treatment patients were followed up for a median period of 27 months, during which different observations, including local recurrence, regional recurrence, lobar recurrence, distant recurrence, and death were recorded. In this work, we have focused on four different outcomes: (1) Overall survival (OS), which is defined as the time from the SBRT to the date of the death or final follow-up visit; (2) Recurrence free survival (RFS), referring to the time from SBRT to the earliest of recurrence (local, lobar, regional, or distant), second cancer, death or final follow-up visit; (3) Local control (LC), defined as the absence of local (within the area of the planning target volume) recurrence, and; (4) Distant control (DC), calculated as the absence of recurrence outside of local, lobar or regional recurrences. There are patients who have more than one lung tumor. Since the outcomes of OS, DC, and RFS are related to the patient and not to each single tumor, we decided to take the tumor with the highest SUV. However, LC is tumor-related, and therefore, all the 150 tumors are treated as data instances.

**Table 4:** Datasets used in the literature for lung cancer time-to-event outcome predictions.

Reference	Number of patients	Difference with our dataset	Availability
Wu et al.^5^	101	Only PET images are utilized and outcome is distant metastasis	Not public
Pyka et al.^6^	45	–	Not public
Huang et al.^7^	282	Only CT images are utilized and outcome is disease-free survival	Not public
Sun et al.^16^	422	Only CT images are utilized and outcome is overall survival	Public^10^
Khorrami et al.^18^	125	Only CT images are utilized and outcome is overall survival and time to progression	Not public
Wang et al.^36^	129	Only CT images are utilized and outcome is overall survival	Not public
Xu et al.^37^	179	Only CT images are utilized and patients are treated with chemoradiation	Not public

All the images are cropped based on the annotations provided by our experienced Radiologist to only contain the tumor region. As the proposed DRTOP model requires fixed-sized inputs and tumors have different sizes, we have zero-padded the cropped tumor regions following the common practice for standardizing the size of inputs to a deep learning architecture. More specifically, cropped tumors are placed in the middle of a black image (intensity of zero), whose size is determined based on the largest tumor available in the dataset. The largest tumor available is of size $80\times 80$ pixels in CT images and $28\times 28$ pixels in PET images. All the images are, therefore, first cropped to completely fit the tumor. Then, cropped CT scans are placed in $80\times 80$ black images, whereas cropped PET scans are placed in $28\times 28$ black images. Determining the size of the inputs, based on the largest tumor, to ensure all the target area is covered, is a standard practice in deep learning-based cancer image analysis^57^. As the inputs to our model are 3D images, where the third dimension is of size 3, three cropped slices, for each tumor, are stacked together. The middle image is the tumor middle slice, and the other two are the two immediate neighbors of the middle slice. At the end, each patient/tumor is associated with two 3D inputs, one generated from the PET component, and the other generated from the CT component.

**Figure 11:**
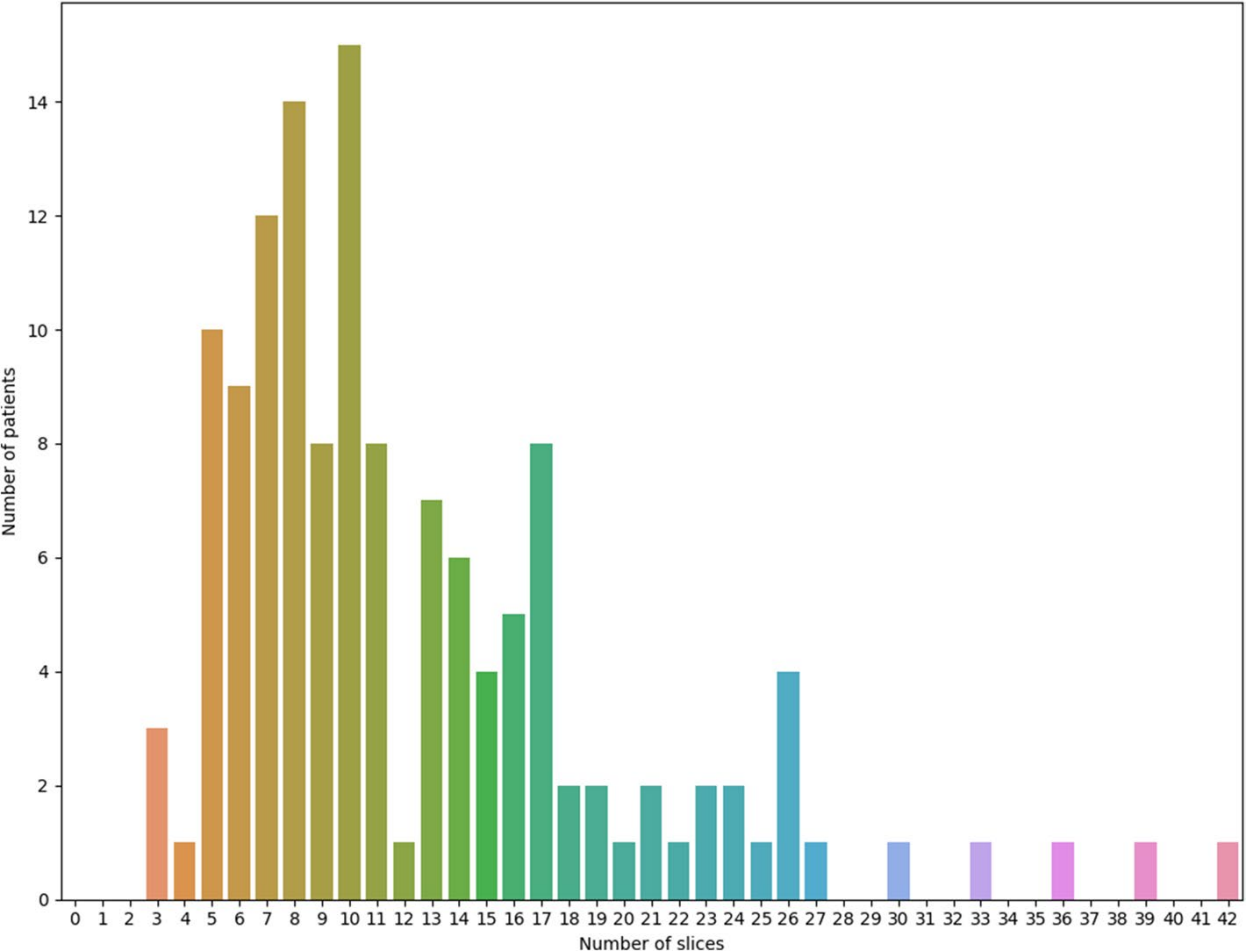
Frequency of number of tumor-containing slices, that can vary from 3 to 42.

Here we further elaborate on the choice of a 3-channel input. As shown in Fig. 11, number of the tumor-containing slices, in our in-house dataset, significantly varies from one patient to another (between 3 and 42). The proposed DRTOP architecture requires a fixed-size input. This means that in case of selecting a higher number of channels, all inputs having less number of tumor-containing slices, have to be accompanied with healthy slices, in order to maintain a fixed size. Accordingly, selecting a higher number of channels leads to the following two important challenges: (1) First, it requires advanced memory resources, and; (2) Second, it makes some tumors too small to be distinguished from surrounding tissues^41^. Furthermore, the 3-channel input has been previously investigated in several studies, leading to satisfying results. For instance, in Reference^42^, 3-channel CT scans are used to predict short and long-term survival in lung cancer, using CNNs, and it has been shown that the 3-channel input outperforms the single channel one. The 3-channel input is also utilized in References^43^ and^44^, for classifying breast tumor and mediastinal lymph node metastasis of lung cancer, respectively, using CNNs.

In order to validate our model and also the hand-crafted method, we have split the dataset into two independent set of training ($80\%$) and testing ($20\%$) instances. The training dataset is used to train our proposed model, and also the hand-crafted method, whereas the test set remains unseen during the training, and is used at the end for evaluating the models.

It is worth mentioning that for lung cancer survival analysis, large datasets are scarce and very difficult to acquire, as patients need to be followed up for years. Studies investigating the problem of lung cancer time-to-event outcome prediction, a few of which are listed in Table 4, therefore, evaluate their models on relatively small datasets. In order to evaluate the proposed DRTOP model, dataset needs to include both PET and CT images that are contoured by an expert, which limits us to the in-house dataset with 132 patients. Furthermore, outputs of the model, i.e., OS, RFS, LC, and DC, are required to be available for all the patients in the dataset. As we have shown in Table 4, the dataset used in Reference^6^ is the only one that includes all the DRTOP’s requirements. This dataset, however, is not publicly available and is limited to 45 patients. To the best of our knowledge, the NSCLC-Radiomics dataset^10^ is the only publicly available data that focuses on the lung cancer survival analysis. Nevertheless, it is accompanied with only CT images and the outcome is limited to the overall survival.

### CNN architecture of the $\text {DRTOP}$ model

CNNs^24^, usually, consist of three type of layers. The core layers are the convolutional layers, applying trainable filters with shared weights on the input. Shared weights help the network to detect local features such as curves and edges across the input. The second important layers are pooling layers, which sub-sample the input, with the intuition that the exact location of the features do not contribute to the final prediction. The third layers are fully connected ones, with the same functionality of a simple neural network. The CNN architecture we used in this work is shown in Fig. 1. We have adopted two separate networks, for CT and PET components, each of which contains two convolutional layers (with $3\times 3$ filters, 32 feature maps, and rectified linear units), two pooling layers (with $2\times 2$ filter size) and two fully connected (FC) layers. The first and second FC layers contain 32 and 1 neurons, respectively. While the first FC utilizes rectified linear units, the second one has a linear activation. Both CNNs are trained separately with the goal of maximizing the Cox partial likelihood. The optimization method is a stochastic gradient descent (SGD), with a learning rate of $10^{-5}$. Number of epochs is set to 2, 000, and while most of the studies on deep learning-based time-to-event analysis feed the model with the whole dataset at once, we used a batch size of 32^45^, to prevent the network from over-fitting the training set. The outputs of the last fully connected layers are treated as radiomics signature (risk), and fed to a Cox PHM, along with the other clinical factors (age, gender, SUV, and radiation dose).

One problem associated with CNNs is that they, typically, require large datasets to be able to learn a useful mapping from the input to the output. Otherwise, the network over-fits the training set, leading to poor predictions for the test set. Large dataset is, however, difficult to collect in the medical field. One solution to compensate for the lack of large dataset is to pre-train the model with, preferably, a similar dataset^46^. Pre-training helps the network with learning the data distribution. Consequently, when training the model supervisedly on the main dataset, weights are initialized by the pre-trained values instead of the random ones, getting one step closer to the optimal solution. The convolutional auto-encoder (CAE)^47,48^, we adopted in this work for the pre-training, will be explained in the next sub-section.

### Pre-training with convolutional auto-encoders

Auto-encoders are unsupervised neural networks that are only fed with the input, without any additional information or labels. The network is aimed to learn features from the input that are useful in reconstructing the input. Auto-encoders consist of two main components, i.e., the encoder, which learns features from the input, and; the decoder, which uses learned features to reconstruct the input. The CAEs are variants of the original Auto-encoders with embedded Convolutional layers, making them powerful models for unsupervised training of the image inputs. In this work, two separate CAEs are trained on the PET and CT components, where the encoder’s architecture is exactly the same as the main CNN architecture described in the previous sub-section. The CNNs are, consequently, initialized with the weights learned in the CAEs, through the unsupervised training.

The dataset we used for the unsupervised pre-training is different from the main dataset. However, it includes pre-treatment PET/CT images of 86 lung cancer patients from a previous work^49^. This dataset does not contain the time-to-event outcomes. Images in this dataset are also annotated by our thoracic radiologist (A.O.), and pre-processed using exactly the same approach as the one used for pre-processing the main dataset.

### Cox proportional hazards model (PHM)

The DRTOP model was trained, separately, for all four outcomes, and calculated the CT and PET risks. These two risks, along with four clinical factors are entered into the Cox PHM, using a stepwise selection of the variables. In other words, the final model includes only the significant predictors, where significance is evaluated based on an F-test of the obtained coefficients. Therefore, to assess the significance of a coefficient, the Cox PHM is trained after excluding the underlying variable (restricted model), and compared against training the model, including all the variables (unrestricted model). The significance level is set to 0.05, and only the variables associated with *p*-values less than the significance level remain in the model. Table 1 shows the four time-to-event outcomes along with their significant predictors. Hazard ratio (HR) measures the effect of the predictors on the outcome. Concordance index^50^, presenting the quality of ranking, is also computed for all the four outcomes. The PHM formulation, based on our predictors is as follows

1 $$\begin{aligned} h(t|x_i) = h_0(t)\exp ^{\big (\beta _1\times CT_i ~+~\beta _2\times PET_i ~+~ \beta _3\times Age_i ~+~ \beta _4\times Gender_i~+~ \beta _5\times SUV_i ~+~ \beta _6\times Dose_i\big )} \end{aligned}$$

where $h(t|x_i)$ refers to the hazard at time *t* for the *i* *th* patient. Term $h_0(t)$ is the base-line hazard, and $\beta _i\text {s} ~(1\le i \le 6)$ are the coefficients (covariates) to be learned with the objective of maximizing the Cox partial likelihood.

It is worth mentioning that in design of the proposed DRTOP model, we have chosen to use the final deep learning output as the inputs to the Cox PHM. The rationale behind this design is to prevent the 64 features, extracted from the layer before the final one, from dominating the Cox PHM, and cancel out potential effects of the clinical factors (age, gender, SUV, and radiation dose). The incorporated strategy is similar in nature to the approach adopted in Reference^18^, where a Least Absolute Shrinkage and Selection Operator (LASSO) Cox model is, first, used to extract the most important radiomics features, before going through the final Cox PHM, along with other clinical factors.

### Random survival forest (RSF)

An RSF model^38^ is a collection of several survival trees, each of which is constructed using a randomly drawn sample of the data and underlying variables. Each survival tree is separately trained, based on a logrank splitting rule, which tries to maximize the survival difference between the daughter nodes. While each tree outputs a separate CHF for each patient, the final outcome is the ensemble CHF. The Nelson-Aalen estimator is used to calculate the CHF, denoted by ${\hat{H}}$, at each terminal node *h*, and is given by

2 $$\begin{aligned} {\hat{H}}_h(t)=\sum _{t_{l,h}\le t}\frac{d_{l,h}}{Y_{l,h}}, \end{aligned}$$

where $t_{l,h}$ denotes a distinct event time at node *h*, and $d_{l,h}$ and $Y_{l,h}$ are number of death and patients at risk, respectively, at time $t_{l,h}$. In this study, the RSF model consists of 10, 000 survival trees. The maximum depth is set to 10, and the minimum node size is 10. To obtain the important predictors, a variable importance (VIMP)^38^ approach is adopted. Based on this approach, for each variable, the prediction error is calculated for the original RSF, and also an RSF with random assignment, when encountering the underlying variable. The VIMP is then calculated as the difference between these two errors. A large positive VIMP indicates a high predictive ability, whereas a zero or negative one means no prognostic value.

**Table 5:** Hand-crafted features, extracted in this work.

Category	Description	Sub-category
First order radiomics	Distribution of pixel intensities and ROI shape	
Shape features	Quantify the geometric shape of the tumor^51^	Area regularity (1), perimeter regularity (2), region bilateral symmetry (4)
Intensity features	Derived from a single histogram^51^	Size of the tumor (number of pixels), mean gray level, standard deviation, median gray level, minimum pixel intensity, maximum pixel intensity, kurtosis, skewness^51,52^
Second order radiomics (Texture features)	Relations between pixels to model intra-tumor heterogeneity^51^	Contrast, energy, correlation, homogeneity, entropy, normalized entropy

### Hand-crafted radiomics

To compare the ability of our proposed $\text {DRTOP}$ model in predicting the time-to-event outcomes, in lung cancer patients, with the hand-crafted radiomics, we have extracted 42 features from the CT and PET components. As shown in Table 5, these features include both first and second-order radiomics, where the former refers to features extracted mostly from the image histogram, and the latter refers to texture-related features. The “Sub-category” column presents the features we have extracted, where the numbers in the parenthesis indicate the number of features extracted from that specific category. All the features, consequently, go through a PCA, where a total of 18 features are extracted. These features are the inputs to a stepwise Cox PHM.

All deep learning methods, used in this study, are implemented using Python 2.7 and the Keras library^53^. The Cox PHM and Kaplan–Meier analysis are carried out using the Python Lifelines library^54^, statistical tests are performed in IBM SPSS Statistics software^55^, and the RSF model is implemented using the PySurvival^56^ library.

### Concordance index

Concordance index (c-index) is a measure of how well the patients are ranked based on a specific time-to-event outcome. Mathematically, it can be defined as^58^

3 $$\begin{aligned} c=\frac{1}{|\xi |}\sum _{T_i~uncensored} \sum _{T_j>T_i}1_{f(x_i)<f(x_j)}, \end{aligned}$$

where $|\xi |$ denotes the number of possible ordered pairs, $T_i$ and $T_j$ are the time-to-event outcomes for Subjects *i* and *j*, respectively, and $f(x_i)$ is the predicted time for Subject *i*. The c-index varies between 0 and 1, where a c-index of 1 means a perfect prediction, and a c-index of 0.5 can be interpreted as a random assignment. In biomedical applications and in particular lung cancer survival analysis, a c-index close to 0.7 is considered as satisfying and acceptable^59,60^, however, interpretation of the computed c-index value depends on the dataset and the problem at hand.

## Data Availability

The datasets generated and/or analyzed during the current study are not publicly available due to the confidentiality restrictions imposed by the approved ethics of study but are available from the corresponding author on reasonable request.
